# Mechanical Performance and Microstructural Characterization of PET-Modified Cement Mortars with Metakaolin

**DOI:** 10.3390/ma19091682

**Published:** 2026-04-22

**Authors:** Aleksandra Kostrzanowska-Siedlarz, Tomasz Ponikiewski, Agnieszka Kocot, Oldrich Sucharda

**Affiliations:** 1 Department of Building Processes and Building Physics, Faculty of Civil Engineering, Silesian University of Technology, 44-100 Gliwice, Poland; aleksandra.kostrzanowska-siedlarz@polsl.pl (A.K.-S.); a.koziolek@onet.eu (A.K.); 2 Department of Building Materials and Diagnostics of Structures, Faculty of Civil Engineering, VSB-Technical University of Ostrava, 708 33 Ostrava-Poruba, Czech Republic; oldrich.sucharda@vsb.cz

**Keywords:** metakaolin, PET flakes, interfacial transition zone, cement mortars, microstructure, mechanical properties, SEM analysis, recycled aggregates

## Abstract

**Highlights:**

- Metakaolin (10 wt%) restores the compressive strength of PET-modified mortars to reference levels.
- SEM analysis confirms that metakaolin densifies the ITZ and reduces interfacial debonding gaps.
- High cement replacement (50 wt%) may lead to strength loss, potentially associated with self-desiccation and autogenous shrinkage.
- Pozzolanic activity of MK improves stress transfer between PET flakes and the cementitious matrix.
- Matrix refinement is essential to mitigate the hydrophobic effects of recycled plastic aggregates.

**Abstract:**

The incorporation of plastic waste into cement-based materials offers a promising strategy for improving sustainability; however, it is often associated with reduced mechanical performance due to weak interfacial bonding. This study investigates the effect of metakaolin on the interfacial transition zone (ITZ) and mechanical properties of cement mortars modified with polyethylene terephthalate (PET) flakes used for the partial replacement of natural sand. Mortars containing 10 and 50 wt% metakaolin (as cement replacement) and 5 vol.% PET flakes (as sand replacement) were prepared and tested after 28 days of curing. Compressive and flexural strength were evaluated, and microstructural analysis was conducted using scanning electron microscopy (SEM) with a focus on the ITZ. The results indicate that the incorporation of PET flakes leads to a reduction in mechanical properties due to the formation of a porous and weak ITZ. However, the addition of 10 wt% metakaolin significantly improved mechanical properties, enabling PET-modified mortars to achieve strength comparable to the reference mix. SEM observations revealed that metakaolin contributed to the refinement of the microstructure and reduction in ITZ porosity, which enhanced interfacial bonding and improved stress transfer between PET particles and the cement matrix. These findings demonstrate that metakaolin can effectively mitigate the negative effects associated with PET incorporation by improving the microstructural characteristics of the ITZ, thereby enhancing the performance of sustainable cement-based composites.

## 1. Introduction

The increasing demand for construction materials and the associated environmental impact, particularly CO_2_ emissions from cement production, have become major global concerns [1,2]. The cement industry is responsible for a significant share of anthropogenic CO_2_ emissions, which has led to intensive research on reducing the clinker content in cement-based materials [1,3,4,5,6]. One of the most effective approaches is the partial replacement of Portland cement with supplementary cementitious materials (SCMs) or alternative binders [3,4,5,6].

Metakaolin (MK), obtained by calcination of kaolinitic clay at temperatures significantly lower than those required for Portland cement production, is widely recognized as an effective supplementary cementitious material in both Portland cement and alternative binder systems [7]. Its application can reduce CO_2_ emissions while improving mechanical properties and durability of cementitious materials due to its high pozzolanic activity [6,8]. The high pozzolanic activity of MK, often enhanced by the presence of reactive silica compounds, contributes to the acceleration of hydration processes and the formation of a more homogenous binder structure. Previous studies have shown that replacing Portland cement with metakaolin at levels between 5 wt% and 30 wt% leads to an increase in compressive strength due to the formation of additional C–S–H phases and refinement of the pore structure [9,10]. However, higher replacement levels may result in increased water demand and porosity, which negatively affect mechanical performance [11,12]. Moreover, the mechanical behavior of metakaolin-modified systems is strongly influenced by curing conditions, particularly temperature, which affects the development of the microstructure and strength [13]. The high reactivity of metakaolin contributes to the refinement of the pore structure and improved microstructural development of cement-based materials [14].

In parallel, increasing attention has been given to the reuse of plastic waste in construction materials. The accumulation of plastic waste, especially polyethylene terephthalate (PET), poses a serious environmental challenge [15,16,17,18]. One promising solution is the incorporation of PET waste as a partial replacement of natural aggregates in cement-based composites [19]. However, numerous studies have reported that the addition of PET particles generally leads to a reduction in mechanical strength, particularly at higher replacement levels [20,21,22,23,24,25,26]. Similar trends have also been observed in alternative binder systems, such as alkali-activated materials, where the incorporation of PET flakes led to a reduction in mechanical performance due to weak interfacial bonding [27].

The reduction in strength is mainly attributed to the weak interfacial bond between PET particles and the cement matrix, as well as the formation of voids and microcracks in the interfacial transition zone (ITZ) [22,24]. Although some studies have shown that small amounts of PET may not significantly affect or may even slightly improve compressive strength [24,25,26], the overall performance of PET-modified mortars remains limited by poor interfacial adhesion.

To overcome this limitation, the use of highly reactive SCMs such as metakaolin may be beneficial. Due to its pozzolanic reactivity, metakaolin can densify the microstructure and refine the ITZ, potentially improving the bond between aggregate particles and the cement matrix [6,25].

Previous studies have investigated the incorporation of PET waste in cementitious materials over a wide range of replacement levels, typically from 1 to 20% of fine aggregate volume [20,21,22,23,24,25,26]. While small amounts of PET may have a limited or even slightly positive effect on compressive strength, higher replacement levels generally result in a significant reduction in mechanical performance. The primary mechanisms responsible for this deterioration include the hydrophobic nature of PET, which leads to poor adhesion with the cement paste, as well as the formation of a weak and porous interfacial transition zone (ITZ). This results in reduced stress transfer efficiency and promotes microcrack initiation and propagation under load.

Various approaches have been proposed to mitigate these effects, including surface treatment of PET particles, optimization of particle size and shape, and the use of supplementary cementitious materials (SCMs) to refine the microstructure. However, the effectiveness of these strategies remains dependent on the specific material system and mixture design. Among the available SCMs, metakaolin (MK) is particularly promising due to its high pozzolanic reactivity, fine particle size, and ability to significantly refine the pore structure and interfacial regions. Compared to other commonly used SCMs such as fly ash or ground granulated blast furnace slag, MK reacts more rapidly and contributes to early-age strength development as well as microstructural densification. Although both PET-modified mortars and MK-blended systems have been widely studied independently, there is still limited understanding of their combined effect, particularly with respect to the modification of the interfacial transition zone and its role in mechanical performance. Most existing studies focus primarily on macroscopic properties, while the microstructural mechanisms governing the interaction between PET particles and the modified cement matrix remain insufficiently explored.

To the best of the authors’ knowledge, the combined effect of PET flakes and metakaolin on the ITZ in cement mortars has not been sufficiently explored in the existing literature. Therefore, the aim of this study is to investigate the influence of metakaolin on the mechanical performance and microstructural characteristics of PET-modified cement mortars, with a focus on the interfacial transition zone. The study evaluates mechanical properties (compressive and flexural strength) and provides microstructural insights with a focus on the ITZ using SEM and EDS analysis.

## 2. Materials and Methods

The studied mortars were designed to evaluate the combined effects of metakaolin as a partial replacement of Portland cement and PET flakes as a partial replacement of natural aggregate.

The material used in this study is a commercially available metakaolin-based additive (provided by Poraver, Schlüsselfeld, Germany) containing pulverized glass, which acts as a pozzolanic micro-filler and contributes to the overall density and reactivity of the binder system. For simplicity, this material is referred to as metakaolin (MK) throughout the manuscript. Its dry loose bulk density was 350 ± 150 kg/m^3^. The chemical composition is shown in Table 1. The chemical composition was provided by the manufacturer.

Portland cement type CEM I 42.5 R was produced by Góradże (Chorula, Poland). Its chemical composition and basic physical properties are shown in Table 2. The bulk density was 3110 kg/m^3^ [28]. The specific surface area of the MK was over 10 times larger than in the case of the used cement. Therefore, higher MK reduced the workability of fresh mortars.

The mix designs used are presented in Table 3. The reference mortar (REF) was prepared according to the EN 196-1 standard [29], with a fixed water-to-cement (w/c) ratio of 0.50 and a sand-to-cement ratio of 3:1. Specifically, each batch consisted of 450 g of cement, 1350 g of standard quartz sand, and 225 g of water. In the modified mixtures, metakaolin (MK) was used as a partial replacement of cement (10 and 50 wt%), while PET flakes replaced 5 vol.% of the natural sand. The amount of water was kept constant for all mixtures to isolate the effect of metakaolin and PET flakes on the mechanical and microstructural properties. The used sand was a standard quartz sand (according to the EN 196-1) [29]. No chemical admixtures (e.g., superplasticizers) were used in this study in order to isolate the effect of metakaolin and PET flakes on the properties of the mortars.

Polyethylene terephthalate was delivered by the recycling company in the form of flakes. PET flakes were derived from beverage bottles, mainly originally containing water. No chemical treatment of PET was applied in order to isolate the effect of metakaolin on ITZ modification. The PET flakes were washed with water. The specific density was 1.38 g/cm^3^. The maximum size of PET flakes was limited to 4 mm to minimize the influence of particle size on the mechanical properties.

Samples were prepared according to the PN-EN 1015-11:2001/A1:2007 [30]. Each test was performed on at least three specimens, and the reported values represent the mean, with standard deviation provided in the corresponding tables. Samples were cast into beams having dimensions of 160 × 40 × 40 mm. All beams were removed from molds after 24 h and stored in water at 20 ± 2 °C for 27 days. Subsequently, all samples were weighed and tested for flexural and compressive strength values. Due to the observed reduction in mechanical performance of mortars containing 50 wt% metakaolin under standard curing conditions, additional samples were cured in sealed plastic bags at 20 ± 2 °C for 27 days.

Beams were cut into cubes (dimensions ranging from 1 to 2 cm) for the Scanning Electron Microscope (SEM) examination. Cubes were stored in alcohol to remove water. Later samples were placed in vacuum for one hour and impregnated with a low viscosity resin under vacuum using the Struers Citovac unit (Ballerup, Denmark). After one hour, samples remained for 24 h at room temperature (20 ± 2 °C) before being removed from molds.

All samples were polished using the Struers LaboForce-100 (Ballerup, Denmark) equipped with an oil dispenser which has been used as cooling and lubricating agent. The grinding procedure was as follows: the grinding plates were sprayed with diamonds having with particle sizes ranging from 1 to 9 µm. The microstructure of polished samples was studied using Scanning electron microscope (SEM) type JSM-IT100, Jeol (Tokyo, Japan). The SEM images were obtained in the Backscattered Electron Composition (BEC) mode at high vacuum, using the accelerating voltage of 6.00–10.0 kV and the probe current 50 mA. The used magnifications ranged from 800 to 1000× magnification.

## 3. Results and Discussion

### 3.1. Physical and Mechanical Properties

**Figure 1:**
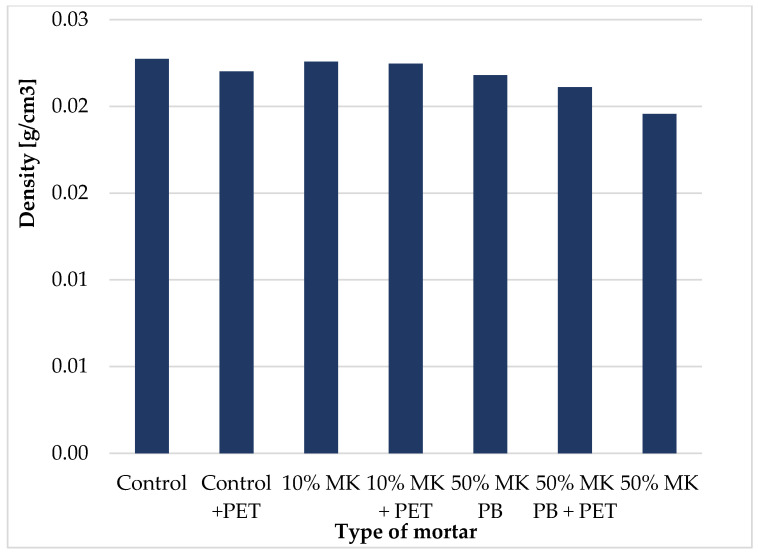
Bulk density of mortars after 28 days of curing, illustrating the influence of metakaolin content and PET flakes incorporation on density. The data represent mean values; detailed numerical results and standard deviations are provided in Table 4.

The densities of mortars determined after 28 days of curing are presented in Figure 1. The bulk density values are illustrated in Figure 1, with precise numerical data and standard deviations detailed in Table 4. The low variability of results confirms the consistency of the specimen preparation process. The incorporation of PET flakes slightly reduced the density of the mortars, particularly in mixtures containing 50 wt% of metakaolin. This reduction is associated with the lower density of PET compared to natural aggregates as well as to the formation of additional voids associated with weak interfacial bonding. Similarly, replacing 50 wt% of cement with metakaolin resulted in a density decrease of 7 wt% and 14 wt% for samples cured in plastic bags and water, respectively, which may be related to increased porosity of the system.

**Figure 2:**
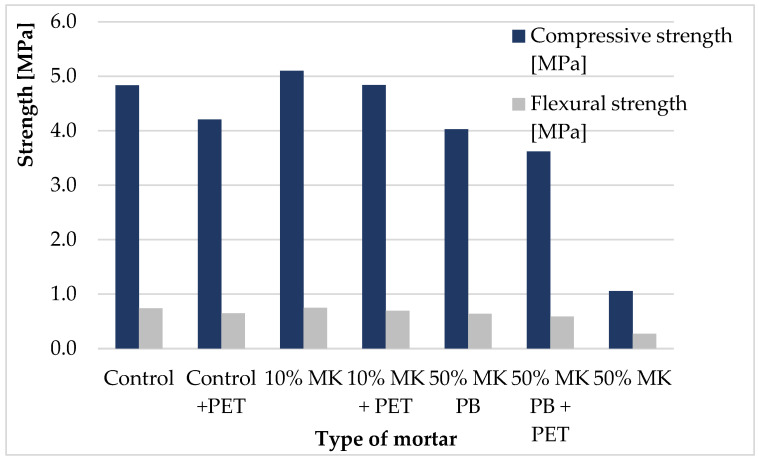
Mechanical properties (compressive and flexural strength) of mortars after 28 days of curing, showing the influence of metakaolin content and PET incorporation. The data represent mean values; detailed numerical results and standard deviations are provided in Table 5.

Mechanical properties of the mortars after 28 days are shown in Figure 2. Due to the high consistency of the mechanical test results (standard deviation below 4.0% of the mean values), the average values are presented in Figure 2, with detailed statistical dispersion provided in Table 5. These data correlate with the visual trends shown in Figure 2.

The results indicate that the incorporation of PET flakes generally led to a reduction in both compressive and flexural strength. This effect is associated with the weak interfacial transition zone (ITZ) formed between PET particles and the cement matrix, which limits stress transfer and promotes microcrack initiation.

The addition of 10 wt% of metakaolin increased compressive strength by approximately 6%, while higher replacement levels (50 wt%) resulted in a significant reduction in strength. However, in mixtures containing both metakaolin (10 wt%) and PET flakes (5 wt%), mechanical properties comparable to the reference mortar were achieved. This suggests that metakaolin can partially compensate for the negative effect of PET incorporation.

The improvement in mechanical performance is associated with the refinement of the microstructure and modification of the ITZ. The pozzolanic reaction of metakaolin leads to the consumption of portlandite and formation of additional C–S–H phases, resulting in a denser binder matrix and improved interfacial bonding.

The significant reduction in mechanical performance observed for mortars with 50 wt% metakaolin may be associated with the specific hydration kinetics of high-MK systems. High replacement levels can lead to self-desiccation due to the rapid consumption of water during the pozzolanic reaction, which may result in autogenous shrinkage and the formation of microcracks within the cement matrix. In mixtures cured without protective sealing, the lack of internal humidity control may further intensify these effects, potentially leading to structural degradation and reduced load-bearing capacity of the composite. However, it should be noted that no direct measurements of shrinkage or internal humidity were performed in this study, and therefore this explanation remains a plausible interpretation based on literature data. Overall, the results indicate that excessive metakaolin content (50 wt%) is not beneficial under the conditions investigated.

The incorporation of PET flakes as a partial replacement of natural sand led to a reduction in the mechanical performance of mortars, primarily due to the formation of a weak interfacial transition zone (ITZ) between the PET particles and the cement matrix. The addition of metakaolin significantly improved the performance of PET-modified mortars, particularly at a 10 wt% cement replacement level, where mixtures containing 5 vol.% PET achieved mechanical properties comparable to or slightly exceeding those of the reference mortar. The obtained results indicate the potential applicability of PET–metakaolin mortars in non-structural or low-load construction elements.

The improvement in mechanical performance is associated with the refinement of the microstructure and modification of the ITZ. The pozzolanic reaction of metakaolin leads to the consumption of portlandite and formation of additional C–S–H phases, resulting in a denser binder matrix and improved interfacial bonding. This effect is consistent with previous studies reporting that metakaolin significantly enhances matrix densification and interfacial bonding due to its high pozzolanic activity and fine particle size [14,25].

It should be noted that maintaining a fixed water-to-cement (w/c) ratio of 0.50 for all mixtures significantly influenced the fresh-state performance. The high specific surface area of metakaolin (over 10 times that of cement) led to a noticeable reduction in workability, particularly in the 50 wt% MK series. This reduced flowability likely resulted in less efficient compaction and a higher content of entrapped air, which—alongside the microstructural self-desiccation—contributed to the observed reduction in mechanical strength. It should be noted that the constant w/c ratio used in all mixtures may have influenced the workability and compaction of the mortars, which could also affect the resulting mechanical properties.

### 3.2. Microstructural Analysis (SEM)

**Figure 3:**
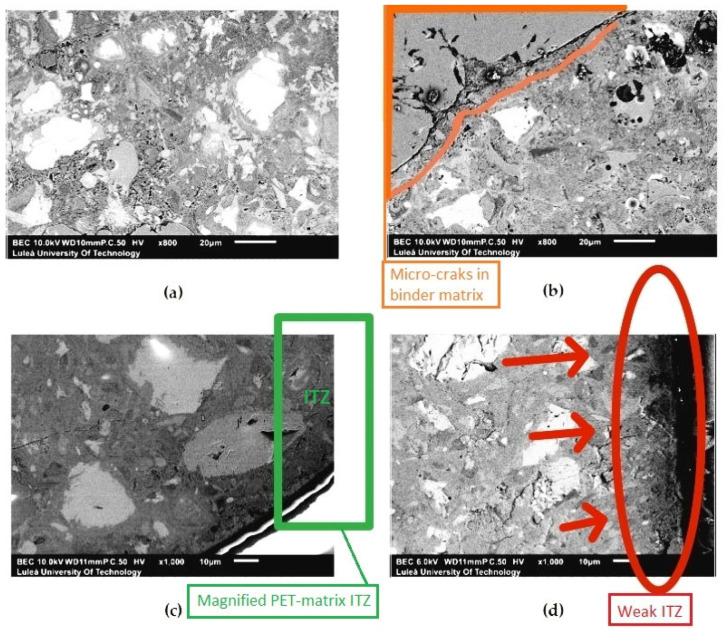
SEM-BSE micrographs illustrating microstructural features of the studied mortars after 28 days of curing: (**a**) 10 wt% metakaolin—relatively dense and homogeneous matrix; (**b**) 50 wt% metakaolin—increased porosity and visible micro-cracking within the binder (orange box); (**c**) 10 wt% metakaolin with PET flakes—green box highlights a region of improved contact between the PET particle and the matrix, indicating local densification of the interfacial transition zone (ITZ); (**d**) reference mortar with PET flakes—clearly visible interfacial gap (red arrows) and localized cracking and porosity (red oval), indicating weak interfacial bonding. Note: Due to the heterogeneous nature of the composite, the ITZ is not uniformly developed; the presented features are representative and illustrate prevailing microstructural trends.

Microstructural observations obtained from SEM analysis (Figure 3) provide qualitative microstructural evidence of the role of the ITZ in controlling the mechanical performance of the studied mortars. The presented images are representative of the observed microstructural features across multiple analyzed regions. In this study, the analyzed ITZ refers specifically to the interface between PET particles and the cementitious matrix, as the conventional ITZ between natural aggregates and the cement paste was not the primary focus of this investigation. The microstructure of the mortar containing 10 wt% metakaolin (Figure 3a) is characterized by a relatively dense and homogeneous binder matrix with a limited number of large pores. This indicates effective pozzolanic activity of metakaolin, leading to the formation of additional C–S–H phases and refinement of the pore structure. In contrast, the mortar with 50 wt% metakaolin (Figure 3b) exhibits increased porosity and a less compact microstructure, with visible micro-cracks occurring within the binder (indicated by the orange box). The reduced amount of hydration products and the presence of larger voids explain the significant decrease in mechanical strength observed for this mixture. The impact of PET flakes on the composite integrity is most evident when comparing the reference and MK-modified series. The SEM image of the reference mortar (Figure 3d) reveals a clearly defined, wide gap at the interface between the PET flake and the cement paste, characteristic of a weak ITZ. This prominent, continuous debonding gap (highlighted by red arrows) represents a preferential path for crack propagation and confirms poor physical adhesion due to the hydrophobic nature of the plastic. Furthermore, minor cracks and increased porosity are visible in the vicinity of the PET particle (red oval). Conversely, in the 10 wt% MK-modified sample (Figure 3c), the interface appears locally more compact and better integrated. As highlighted by the green box, the PET flake exhibits a smooth, clean edge in direct contact with the densified matrix, confirming the pore-refining effect of metakaolin. However, due to the inherent heterogeneity of the microstructure, the ITZ integrity may vary locally. The pozzolanic reaction of metakaolin contributes to the consumption of portlandite and the formation of additional C–S–H phases, which results in a denser microstructure, particularly within the ITZ region [25]. This enhancement facilitates more efficient stress transfer across the interface by reducing microstructural discontinuities. This effect is consistent with previous studies reporting that metakaolin significantly enhances matrix densification and interfacial bonding due to its high pozzolanic activity [14,25].

Additionally, signs of partial separation at the PET–matrix interface may be further influenced by the potential hydrolysis of PET in the highly alkaline environment, which can lead to the formation of microvoids around the particles [16]. These observations confirm that the mechanical performance of PET-modified mortars is strongly governed by the quality of the ITZ. A moderate addition of metakaolin (10 wt%) effectively mitigates the adverse effects of PET incorporation through localized densification of the ITZ. It should be noted that the metakaolin used in this study contained a minor addition of pulverized glass, which may have contributed to the observed refinement; therefore, the results represent a metakaolin-glass composite system. While the current SEM-BSE analysis is primarily qualitative, the observed reduction in ITZ porosity is consistent with the improved mechanical properties and the chemical evidence provided by EDS analysis (Section 3.3). Consequently, advanced quantitative characterization—such as image binarization, higher-magnification SEM analysis, or nanoindentation—is recommended as a necessary direction for future research to fully quantify these interfacial modifications.

### 3.3. EDS Analysis of MK—Modified Matrix

**Figure 4:**
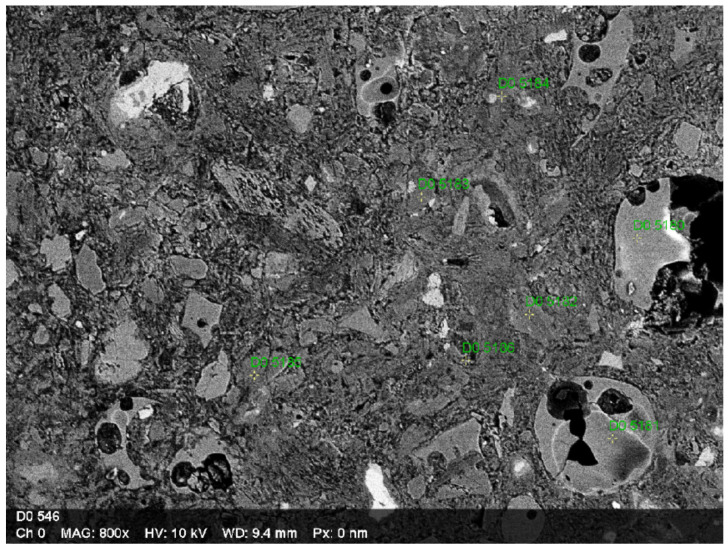
SEM image of the 50 wt% MK mortar matrix with indicated EDS analysis points.

The locations of the EDS analysis points are shown in Figure 4, which complements the qualitative SEM observations presented in Figure 3. To further investigate the microstructural characteristics of the modified binder, an EDS (Energy-Dispersive X-ray Spectroscopy) analysis was performed on the mortar containing 50 wt% metakaolin (MK). During SEM observations, specific particles were identified that were not present in the reference cement matrix. The results of point analysis (Table 6) indicate the presence of particles (points D0 5180 and D0 5181) characterized by elevated concentrations of silicon (Si) and sodium (Na), with minor amounts of aluminum (Al). These features are consistent with the presence of powdered glass introduced as part of the commercial metakaolin-based material. In the analyzed micro-areas, calcium (Ca) was not detected. While calcium is typically present in hydration products of Portland cement systems (e.g., portlandite and C–S–H). The absence of calcium in the selected micro-areas suggests that these regions are dominated by silica-rich phases, likely associated with the presence of glass particles and pozzolanic reaction products. However, due to the localized nature of point EDS analysis, this observation should be interpreted cautiously. These observations may be associated with ongoing pozzolanic reactions and potential modification of the binder composition; however, it should be emphasized that the present EDS analysis is limited to point measurements and does not provide quantitative phase identification. Therefore, the results should be interpreted as qualitative evidence of chemical heterogeneity within the matrix rather than direct proof of specific reaction mechanisms. Nevertheless, the presence of silica-rich phases may contribute to microstructural refinement, which is consistent with the improved mechanical performance observed in mixtures with lower MK content (e.g., 10 wt% MK).

The EDS results support the qualitative SEM observations, indicating that the presence of reactive silica-rich phases contributes to matrix densification and improved ITZ characteristics. Despite limitations, the combined SEM and EDS observations provide consistent qualitative evidence supporting the proposed microstructural trends.

## 4. Conclusions

1. The incorporation of 5 vol.% PET flakes as a sand replacement reduces the compressive strength of the reference mortar by approximately 13.5% (from 46.5 MPa to 40.2 MPa), primarily due to weak interfacial bonding.
2. The addition of 10 wt% metakaolin effectively mitigates this loss, improving the compressive strength of PET-modified mortars by 17.8% compared to the PET-only series, resulting in a performance (46.2 MPa) comparable to the reference mix.
3. High cement replacement with metakaolin (50 wt%) without proper curing conditions results in a significant strength loss of over 58%. This structural degradation is suggested to be related to severe self-desiccation and autogenous shrinkage, typical for high-volume MK systems.
4. Microstructural analysis indicates that metakaolin enhances performance by densifying the interfacial transition zone (ITZ) and reducing visible voids at the PET–matrix interface, thereby improving stress transfer.
5. Untreated PET flakes exhibit signs of surface instability in the highly alkaline environment of the mortar, which may contribute to weak interfacial bonding and further highlights the importance of matrix refinement using reactive SCMs such as metakaolin.
6. While the SEM observations and preliminary EDS analysis provided consistent qualitative and semi-quantitative evidence of microstructural refinement, the authors acknowledge that a detailed quantitative characterization of the ITZ—incorporating techniques such as porosity distribution analysis or microhardness testing—would further strengthen the mechanistic findings. These approaches are considered a vital direction for future research to fully quantify the bond enhancement provided by metakaolin in PET-modified systems.

The results highlight the key role of the interfacial transition zone in governing the performance of PET-modified cementitious composites and demonstrate that metakaolin can effectively improve interfacial properties. These findings provide new insight into the microstructural mechanisms governing the properties of sustainable cement-based materials. Although the SEM analysis was primarily qualitative, the observed trends consistently support the role of metakaolin as an effective modifier of the interfacial transition zone. The study focuses on a single PET content; therefore, the observed trends should be interpreted within this range and may differ for higher replacement levels.

The results contribute to the development of more sustainable cement-based composites by enabling the effective incorporation of plastic waste without significant loss of mechanical performance. The results obtained suggest that mortars containing low amounts of PET and metakaolin may be suitable for non-structural or moderately loaded applications, where sustainability and material efficiency are prioritized.

## Figures and Tables

**Table 1:** The chemical composition of metakaolin according to the producer’s data (provided by Poraver, Schlüsselfeld, Germany).

Chemical Composition of Metakaolin	[%]
SiO_2_	51–63
Al_2_O_3_	19–34
Na_2_O	8.0–10
K_2_O	1.0–2.0
CaO	0.5–5.0
MgO	0.1–2.0
Fe_2_O_3_	0.1–1.0
TiO_2_	0.1–0.5
P_2_O_5_	0.1–0.5
SO_3_	<0.1
Loss on ignition	<1.5

**Table 2:** Properties of metakaolin (provided by Poraver, Schlüsselfeld, Germany) and cement (provided by Góradże, Chorula, Poland) based on producers’ data.

Properties	CEM I	Metakaolin
Bulk density [kg/m^3^]	3110	350 ± 150
Specific Surface [m^2^/g]	0.38	3.5–9.5
Moisture Content	not available	<1.0%
Loss on ignition	2.74	<1.5%

**Table 3:** Mix design of mortars.

Mix	Cement [%]	Metakaolin [wt%]	PET [vol.% of Sand]	Natural Sand [%]	w/c	Curing
Control (REF)	100	0	0	100	0.5	Water
Control + PET	100	0	5	95	0.5	
10% MK	90	10	0	100	0.5	
10% MK + PET	90	10	5	95	0.5	
50% MK	50	50	0	100	0.5	
50% MK PB + PET	50	50	5	95	0.5	Plastic bag
50% MK PB	50	50	0	100	0.5	Plastic bag

**Table 4:** Bulk density of the tested mortars (Mean ± SD).

Mix ID	Bulk Density [g/cm^3^]
Control (REF)	2.27 ± 0.02
Control + PET	2.20 ± 0.03
10% MK	2.26 ± 0.02
10% MK + PET	2.24 ± 0.02
50% MK PB	2.18 ± 0.04
50% MK PB + PET	2.11 ± 0.05
50% MK	1.96 ± 0.06

**Table 5:** Mechanical properties of the tested mortars (Mean ± SD).

Mix ID	Compressive Strength [MPa]	Flexural Strength [MPa]
Control (REF)	46.5 ± 1.2	6.8 ± 0.3
Control + PET	40.2 ± 1.5	5.4 ± 0.4
10% MK	50.8 ± 1.0	7.5 ± 0.2
10% MK + PET	46.2 ± 1.3	6.2 ± 0.3
50% MK PB	30.2 ± 2.2	5.1 ± 0.4
50% MK PB + PET	36.1 ± 2.5	4.9 ± 0.4
50% MK	11.5 ± 3.8	2.5 ± 0.4

**Table 6:** Atomic concentration results from EDS analysis of 50 wt% MK mortar.

Analysis Point	Si (at.%)	Na (at.%)	Al (at.%)
D0 5180	2.96	0.58	-
D0 5181	4.25	0.71	0.68
D0 5182	0.27	-	-
D0 5183	0.56	-	0.22

## Data Availability

The original contributions presented in this study are included in the article. Further inquiries can be directed to the corresponding authors. The data presented in this study are also openly available on Zenodo https://doi.org/10.5281/zenodo.19650167.
